# Core-shell self-assembly triggered *via* a thiol-disulfide exchange reaction for reduced glutathione detection and single cells monitoring

**DOI:** 10.1038/srep29872

**Published:** 2016-07-14

**Authors:** Zhen Zhang, Yuting Jiao, Yuanyuan Wang, Shusheng Zhang

**Affiliations:** 1 Shandong Province Key Laboratory of Detection Technology for Tumor Makers, College of Chemistry and Chemical Engineering, Linyi University, Linyi 276000, China; 2 Collaborative Innovation Center of Functionalized Probes for Chemical Imaging in Universities of Shandong, Shandong Normal University, Jinan 250014, China

## Abstract

A novel core-shell DNA self-assembly catalyzed by thiol-disulfide exchange reactions was proposed, which could realize GSH-initiated hybridization chain reaction (HCR) for signal amplification and molecules gathering. Significantly, these self-assembled products *via* electrostatic interaction could accumulate into prominent and clustered fluorescence-bright spots in single cancer cells for reduced glutathione monitoring, which will effectively drive cell monitoring into a new era.

Reduced glutathione (GSH) is found to be the main nonprotein thiol in cells, which is composed of a tripeptide of cysteine, glycine and glutamic acid^1^,^2^. It plays an essential role in cell metabolism, cell proliferation, cellular homeostasis, and disease resistance^3^,^4^. Glutathione exists in redox equilibrium between disulfide (GSSG, oxidized form) and sulfhydryl (GSH, reduced form) forms, and GSH levels in the living cells alter remarkably in response to the oxidative stress, which has been involved in many diseases and speeds up the aging process^5^,^6^. Under oxidative stress, GSH can be converted into the oxidized form (glutathione disulfide, GSSG) to protect cells from the oxidative stress and assist to capture free radicals that can injure RNA and DNA^7^,^8^,^9^. As a sensitive indicator, any variation of the optimal intracellular ratios of GSH to GSSG can result in human diseases such as cancer, cardiac disease, apoplexy and many neurological disorders^10^,^11^,^12^,^13^,^14^,^15^. Especially in some cancer cells, GSH concentrations in the cytoplasm are generally higher, compared with those of normal cells^12^,^13^. Thus, sensitive detection methods for the analysis of GSH are highly demanded.

Various analytical methods for the GSH determination, such as fluorescence^13^,^14^,^15^, electrochemistry^16^,^17^,^18^, high performance liquid chromatography^19^,^20^,^21^, chemiluminescence^22^ and spectrophotometric assays^23^. Nevertheless, some drawbacks such as derivatization, cumbersome laboratory procedures, time consumption and applicable feasibility *in vivo* were not conducive to the development of a practical method. In this study, a fluorescent-sensing platform based on thiol-disulfide exchange reactions is presented for GSH detection.

Nucleic acids are an essential tool in detection and regulation of gene expression and protein activity for biology and chemistry^24^,^25^,^26^. Main challenges of employing nucleic acids for cell biology and therapy are the efficiency for the sensitive target measure and their intracellular delivery^27^,^28^. Nevertheless, current nucleic acid methods exhibit representatively the restricted sensitivity for living cell imaging and intracellular analysis^29^,^30^,^31^. This defect is primarily ascribed to the lack of signal amplification and collecting signal-molecules mechanisms in these nanotechnology. Recently, mesoporous silica nanospheres (MSNs) as nano-carriers attracted much attention due to chemically modifiable surfaces, high surface area, characteristic nanostructure, biocompatibility and a large load capacity^32^,^33^,^34^. Notably, MSNs with positively charged groups (PCGs) have a unique feature that can adsorb the single-stranded DNAs (ssDNAs) at neutral pH^34^. An interesting potential of MSNs could construct the self-assembled nucleic acid congeries that flexibly adsorbed on the surface of MSNs with positively charged groups, which may be owing to possessing the ssDNAs characteristic of congeries^34^,^35^,^36^.

Inspired by that mentioned above, we proposed a core-shell DNA self-assembly catalyzed *via* thiol-disulfide exchange reactions that could realize target-initiated hybridization chain reaction (HCR) for reduced glutathione detection. Importantly, a non-destructive signal amplification tactic for GSH monitoring in living cells was developed. This core-shell self-assembly had a nuclear nanosphere and an outer layer of fluorogen-labeled nucleic acid congeries, attributed to the HCR products triggered *via* thiol-disulfide exchange reactions. The intracellular core-congeries supplied efficient signal enlargement, fluorescence gathering and enabled super-sensitive monitoring of GSH levels. Moreover, *in situ* imaging of GSH in living cells could boost the monitoring of the distribution and dynamical expression of GSH, and research on GSH-related cellular processes and diseases.

In Fig. 1, a novel core-shell self-assembly method catalyzed *via* thiol-disulfide exchange reactions was presented, which was employed to target GSH and trigger the HCR reaction for intracellular GSH monitoring. A key design was the core-shell DNA self-assembly that could realize GSH-initiated core-HCR for signal amplification and molecules gathering. First, three hairpin-structured DNA probes H_1_, H_2_ and H_3_ were designed, H_1_ including a disulfide bond (-S-S-), H_3_ labeled with a fluorophore/quencher pair. The H_1_, H_2_ and H_3_ probes could be flexible enough to synergistically interact with the positively charged surface, enabling H_1_, H_2_ and H_3_ probes to be steadily assembled on the aminopropyl-modified cores, as core-H_1_H_2_H_3_ probes. Second, after the core-H_1_H_2_H_3_ probes were incubated into living cells, a target GSH was able to fracture the disulfide bond of H_1_ probe *via* a thiol–disulfide exchange reaction. The fractured H_1_ probe initialized the hybridization with H_2_ and generated a single-stranded tail in H_2_, which might dissociate or enhance the mobility of H_2_ on the surface of cores, benefiting its hybridization with H_3_ and renewing a single strand tail in H_3_. Third, by this means, a chain reaction was catalyzed for alternating hybridization between H_2_ and H_3_, creating a chain-like assembly of H_2_ and H_3_^37^,^38^,^39^. These HCR products had a regular conformation, self-assembled on the surface of cores *via* electrostatic interaction, as core-congeries. It could produce a GSH-activity-related fluorescence signal *via* the fluorescence resonance energy transfer, due to the fluorophore (FAM) relatively far away from the quencher (BHQ). Because this unique DNA self-assembly resulted in core-shell nanostructure with a positively charged interlayer and nucleic acid congeries, therefore it could offer high efficiency for cellular delivery^35^ and fluorescence activation.

## Results and Discussion

### Characterization of the Materials

**Figure 2:**
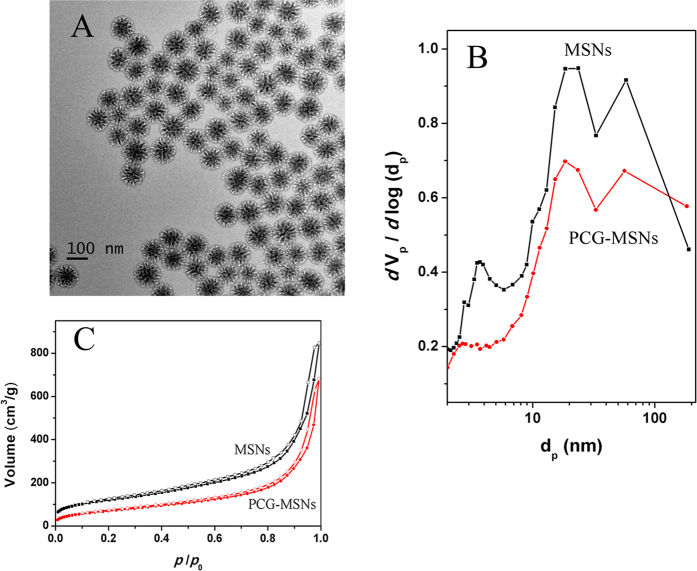
(**A**) TEM image of PCG-MSNs synthesized; (**B**) Pore size distributions of PCG-MSNs and MSNs; (**C**) N_2_ adsorption-desorption isotherms of PCG-MSNs and MSNs.

The employed materials were displayed by transmission electron microscopy (TEM) as shown in Fig. 2A, the average size of them was about 100 nm. PCG-MSNs (preparation in the ESI) and MSNs exhibited similar type of N_2_ adsorption-desorption isotherms. However, it should be pointed out that the amount of the N_2_ adsorbed on the PCG-MSNs was much lower than that adsorbed on the MSNs. And correspondingly, PCG-MSNs possessed the Burnauer-Emmett-Teller (BET) surface area of 262.4 m^2^ g^−1^, which was much smaller than that of the MSNs. From the BJH pore size distributions in Fig. 2, we could find that the pores for the MSNs centered at 4 nm disappeared completely while the pores around 20 nm decreased obviously. These data showed that the massive positively charged aminopropyl groups have been modified into the pores of MSNs.

### Preparation and Cytotoxicitiy Tests of of PCG-MSNs Assembled H1, H2 and H3 Probes

PCGs-MSNs were dispersed in 2 mL hybridization buffer (pH 7.4), and stirred continuously at 37 °C for 90 min to obtain the PCG-MSNs solution (10 mg/ml). The hairpin DNA-H_1_ probe (2 ul 1.0 × 10^−5^ M), the hairpin DNA-H_2_ probe (20 ul 1.0 × 10^−5^ M), the hairpin DNA-H_3_ probe (20 ul 1.0 × 10^−5^ M), 10 ul of 10 mg/ml PCG-MSNs and 100 ul hybridization buffer were mixed in a 1.5 mL Eppendorf tube. After the mixture was stirred continuously at 37 °C for 180 min, the excess reagents were moved away by centrifuging at 10000 rpm for 10 min. The sediment was washed and centrifuged repeatedly for two times to obtain the PCG-MSNs-H_1_H_2_H_3_ probes.

**Figure 3:**
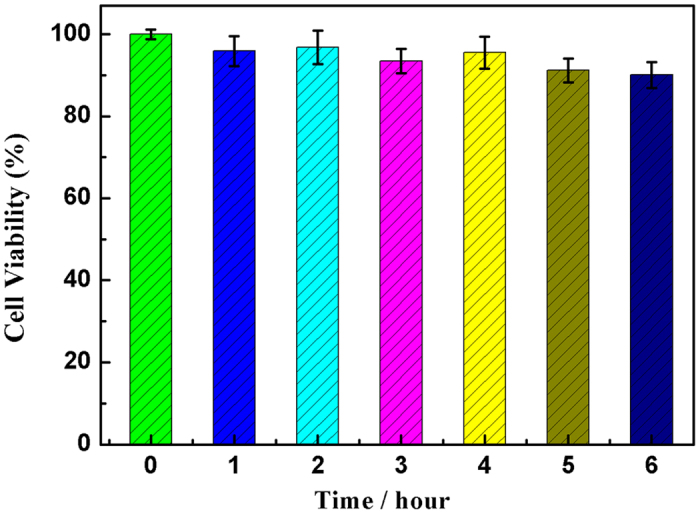
Viability of Hela cells (100 μL, 1.0 × 10^6^ mL^−1^) after incubation with PCG-MSNs-H_1_H_2_H_3_ probes (50 mg/ml) for different times.

The cytotoxicity tests of PCG-MSNs assembled H_1_, H_2_ and H_3_ probes (PCG-MSNs-H_1_H_2_H_3_) were studied with Hela cells by MTT experiment^40^,^41^, as shown in Fig. 3. Briefly, the cells were incubated with 200 μL culture medium containing 15 μL PCG-MSNs-H_1_H_2_H_3_ probes for different times, then were washed with 200 μL hybridization buffer (pH 7.4) once, MTT (0.5 mg mL^−1^, 100 μL) were seeded in the wells and incubated at 37 °C for 4 h. Afterwards, 150 μL DMSO was added to each well to dissolve the crystals constituted by the living cells, and the absorbance at 490 nm was tested to get the relative cell viability. The data showed that Hela cells maintained about 90.3% of the cell viability by (A_test_/A_control_) × 100% after incubation with 30 μL probe for 6 h, revealing that the good biocompatibility.

### Analysis of the HCR Reaction, and Fluorescence Responses of Core-Congeries Dispersion and Liquid Supernatants

**Figure 4:**
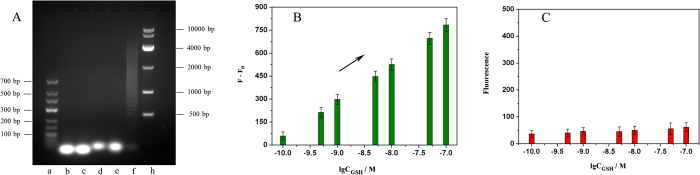
(**A**) The HCR reaction was analyzed by 1.0% agarose gel electrophoresis, the marker (a,h), H_1_ (b), H_2_ (c), the mixture of H_1_ and H_2_ (d), the mixture of H_1_, H_2_ and H_3_ (e), the HCR product (f); Fluorescence responses of core-congeries dispersion (**B**) and liquid supernatants (**C**), GSH concentrations: 0 M, 10^−10^ M, 5.0 × 10^−10^ M, 10^−9^ M, 5.0 × 10^−9^ M, 10^−8^ M, 5.0 × 10^−8^ M, and 10^−7^ M.

The HCR reaction activated via GSH was analyzed via polyacrylamide gel electrophoresis (10%, 110 V, 70 min), stained with ethidium bromide (EB), as shown in Fig. 4A. In stark contrast to fluorescence responses of core-congeries (*F* represent fluorescence intensitiy of amplification products in the presence GSH; *F*_*0*_ represent fluorescence intensitiy in the absence of GSH; *C* represents the concentration of GSH), fluorescence responses of liquid supernatants were very lower (Fig. 4B,C), which hinted that core-congeries produced by hybridization chain reaction could be flexibly adsorbed on the surface of cores.

### Detection Capability for GSH

**Figure 5:**
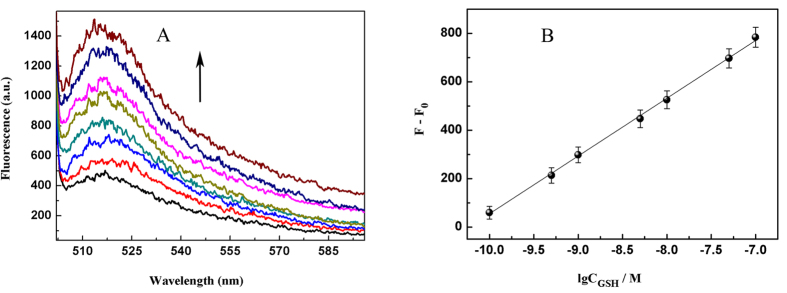
Fluorescence spectral responses to GSH target of varying concentrations *in vitro*. GSH concentrations: 0 M, 10^−10^ M, 5.0 × 10^−10^ M, 10^−9^ M, 5.0 × 10^−9^ M, 10^−8^ M, 5.0 × 10^−8^ M, and 10^−7^ M. (**A**) Core-congeries dispersion; (**B**) The corresponding calibration curve of fluorescence intensity *versus* the concentration of GSH. The average of three spectra was gained from different detection, and three repetitive experiments were implemented. Error bars showed the standard deviation of three experiments. The blank was deducted from each value.

To evaluate the validity of this method, the fluorescence response to different concentrations of GSH in Fig. 5, under the optimum conditions (The detail of the optimal experiments in the ESI). The fluorescence intensities had a fine linear to the logarithm of GSH concentrations in the range from 10^−10^ M to 1.0 × 10^−7^ M in Fig. 5B. The regression equation was depicted as: *F* − *F*_*0*_ = 238.6 l g*C* + 2440.1, the corresponding correlation coefficient (*R*^*2*^) of calibration curve was 0.997, and the detection limit of GSH detection was calculated to be 2.0 × 10^−11^ M (3*σ*). The reproducibility of the core-congeries system was investigated by 11 repetitive measurements of 5.0 × 10^−8^ M GSH under the optimal conditions. The relative standard deviation (RSD) was 11.2%, indicating a fine reproducibility of the method.

### Cell Imaging for Monitoring GSH

**Figure 6:**
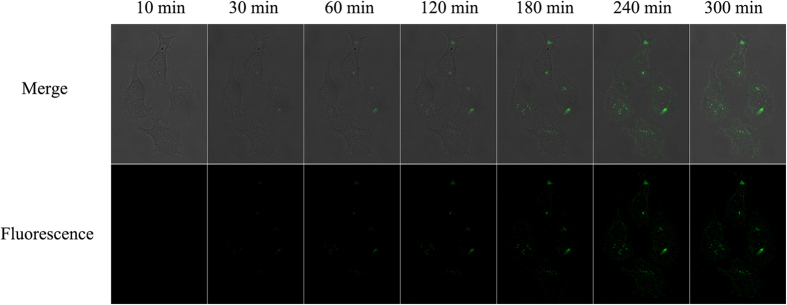
Time course of confocal images of HepG2 cells by the core-HCR method catalyzed *via* a thiol-disulfide exchange reaction.

**Figure 7:**
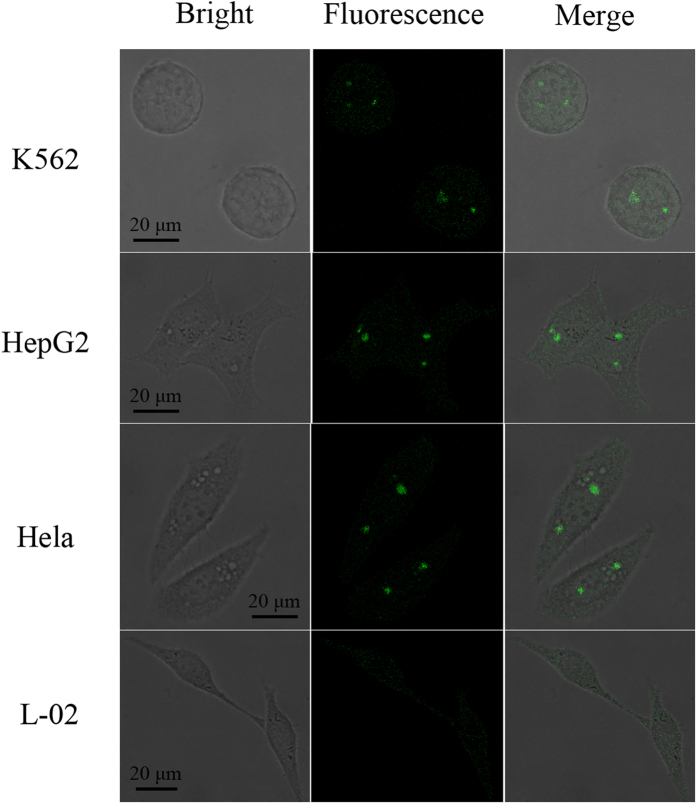
Evaluation of this core-HCR method catalyzed by a thiol-disulfide exchange reaction for target GSH monitoring in living cells: K562, HepG2, Hela cells and human normal hepatocytes.

The response and efficient delivery of the core-congeries nanoassembly *in vitro* offered the possibility for sensitively monitoring of GSH expression in single cells. For the imaging experiments, four types of cells (K562, HepG2, Hela, L-02) were cultured in 6-well slides. In short, the cells were incubated with 700 μL culture medium including the core-H_1_H_2_H_3_ probes (50 mg/ml) for 4 h, then were washed three times with hybridization buffer (pH 7.4). The core-H_1_H_2_H_3_ probes could be rapidly adopted by the cells due to the targeted adsorption and endocytosis, which chiefly led to the higher level of nanospheres internalization into cells within 2 h^42^,^43^. In Fig. 6, upon addition of the core-H_1_H_2_H_3_ probes, the cells did not show observable fluorescence within initial 10 min. After 30 min, fluorescence emerged into the cytoplasm and its intensity increasingly heightened ascribed to the GSH-triggered HCR in living cells. The stronger fluorescence-bright spots revealed that the core-H_1_H_2_H_3_ probes were successfully transported into the living cells, and the core-HCR nano-assembling was achieved in the single cells. The fluorescence intensity was attained the maximum at 240 min (Figure S3). The fluorescence-bright spots in single cells were observed by employing confocal microscopy. The processes of monitoring intracellular GSH were carried out at 37 °C within 4 h, as shown in Fig. 7. The clustered and prominent fluorescence-bright spots could be visualized vividly in living K562, Hela and HepG2 cells, which displayed that low-abundance GSH monitoring in single tumor cells was realizable by this core-HCR method catalyzed *via* a thiol-disulfide exchange reaction. Nonetheless, the big fluorescence-bright spots emerged hardly in human normal hepatocytes (L-02). These data implied that the relative expression levels of GSH in living HepG2, K562 and Hela cells were higher than in L-02 cells. So this novel core-HCR method triggered via a thiol-disulfide exchange reaction was practicable to detect changes in GSH expression levels in single tumor cells.

## Conclusion

This work reported a novel core-shell DNA self-assembly catalyzed by thiol-disulfide exchange reactions, which could realize target-initiated hybridization chain reaction for signal amplification and molecules gathering. Significantly, the self-assembled core-congeries could accumulate into prominent and clustered fluorescence-bright spots in single cancer cells for GSH monitoring. This finding has exciting potential in the study of pathological variations or biological processes in live cells in virtue of its good selectivity and excellent sensitivity.

## Materials and Methods

### Chemicals and Reagents

K562 and Hela cells were purchased from KeyGEN biotechnology Company (Nanjing, China). Human hepatocellular liver carcinoma cell line HepG2 was bought from Shanghai Bioleaf Biotechnology Company (Shanghai, China), and human normal hepatocytes L-02 was from Silver Amethyst Biotech. Co. Ltd. (Beijing, China). MirVana Glutathione isolation kit and Fetal bovine serum were purchased from Life Technologies (Carlsbad, California). Tris-HCl, NaCl, MgCl_2_, EDTA, 3-aminopropyltriethoxysilane (APTES), triethanolamine (TEAH_3_), and tetraethyl-orthosilicate (TEOS) were purchased by Aladdin. All the water used in the work was RNase-free. Hybridization buffer (pH 7.4) contained 10 mM Tris-HCl, 50 mM NaCl, 1 mM EDTA, and 10 mM MgCl_2_. 3-(4,5-dimethyl-thiazol-2-yl)-2,5-diphenyltetrazolium bromide (MTT) and dimethyl sulphoxide (DMSO) were bought from Sigma Chemical Company. Cetyl-trimethylammonium tosylate (CTATos) was purchased from Merck. Unless otherwise mentioned, ultrapure water was used throughout the experiments. All other reagents employed in this work were analytical grade and were used without further purification. All oligonucletides used in the present study were purchased by Sangon Biotech Co., Ltd. (Shanghai China). The sequences were as follows:

H_1_: 5′-TTT TTT- GT CTC TGA GAG TTT -*S-S*- TCT CAA GGA TCA CCG CAT CTC TCA GAG AC-3′

H_2_: 5′-TGA GAG ATG CGG TGA TCC TTG AGA CTA AGT TCT CAA GGA TCA CCG CAT-3′

H_3_: 5′-TCT CAA GGA T -FAM- CA CCG CAT CTC TCA ATG CGG T -BHQ- GA TCC TTG AGA ACT TAG-3′

### Apparatus and Characterization

Fluorescence imaging was performed by a Leica TCS SP8 inverted confocal microscope (Leica, Germany). The cellular images were acquired using a 100× objective. Solid laser (488 nm) was used as excitation source for FAM-labeled probe, and a 495–545 nm bandpass filter was used for fluorescence detection. Transmission electron microscopy (TEM) was measured on a JEOL JEM-2100 instrument. All fluorescence measurements were carried out on a F4600 fluorometer (Hitachi, Japan).

### Cell Culture and GSH Preparation

K562, HepG2, Hela and L-02 cells were respectively cultured in RPMI 1640 (Hyclone, penicillin 100 U/mL, streptomycin 100 μg/mL) adding 10% fetal bovine serum and maintained at 37 °C in a humidified atmosphere including 5% CO_2_, in terms of the instructions of the American Type Culture Collection. K562 cells were elected to be as a representative to analyze the intracellular GSH levels. K562 cells were collected and centrifuged at 3000 rpm for 6 min in a culture medium, washed once with hybridization buffer (pH 7.4), and then spun down at 3000 rpm for 6 min. The cell pellets were suspended in 800 μL of lysis solution.

## Additional Information

**How to cite this article**: Zhang, Z. *et al*. Core-shell self-assembly triggered *via* a thiol-disulfide exchange reaction for reduced glutathione detection and single cells monitoring. *Sci. Rep.* **6**, 29872; doi: 10.1038/srep29872 (2016).

## Supplementary Material

Supplementary Information

## Figures and Tables

**Figure 1:**
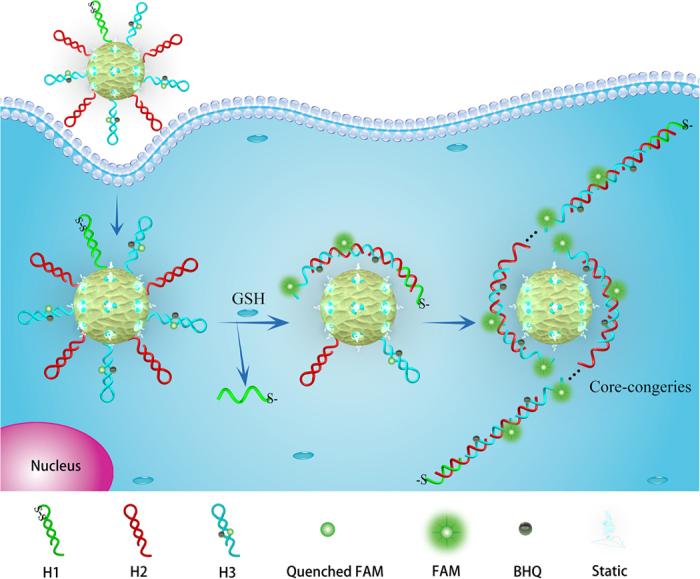
Schematic illustration of GSH detection and single cell monitoring by the core-HCR method catalyzed *via* a thiol-disulfide exchange reaction.
