# Supplementary material for: Core-shell self-assembly triggered via a thiol-disulfide exchange reaction for reduced glutathione detection and single cells monitoring
Source: Sci Rep. 2016 Jul 14;6:29872. doi: 10.1038/srep29872 (PMC4944157; doi:10.1038/srep29872)
Supplement: Supplementary Information [file srep29872-s1.pdf]

Supplementary Information

Core-shell self-assembly triggered *via* a thiol-disulfide exchange reaction for reduced glutathione detection and single cells monitoring

Zhen Zhang,[1] Yuting Jiao,[2] Yuanyuan Wang,[2] Shusheng Zhang [1 *]

[1] Shandong Province Key Laboratory of Detection Technology for Tumor Makers, College of Chemistry and Chemical Engineering, Linyi University, Linyi 276000, China.

[2] *Collaborative Innovation Center of Functionalized Probes for Chemical Imaging in Universities of Shandong, Shandong Normal University, Jinan 250014, China.*

Zhen Zhang (E-mail: zhangzhen19801981@126.com)

Yuting Jiao (E-mail: yuting_jiao@126.com)

Yuanyuan Wang (E-mail: yuanyuan19881989@126.com)

Shusheng Zhang* (E-mail: shushzhang@126.com)

*Corresponding author. Tel: + 86 539 8766107; fax: + 86 539 8766107.

E-mail address: shushzhang@126.com (Shusheng Zhang).

The optimization of experimental conditions

The incubation temperature, pH and time of the reaction solution are three important factors affecting nucleic acids hybridization. For achieving the best sensing performance, a series of control experiments were designed to optimize the incubation temperature, pH and time. Fluorescence intensities enhanced speedily when incubation time increased from 1h to 6h. However, the fluorescence intensity reduced slightly after 4h. So 4h of incubation was considered to be the optimum. Figure S1 showed the influence of pH and temperature on the fluorescence signal measured by 1.0 $\times 10^{-8}$ M GSH. Because of physiological conditions in living cells, 37 ℃ and pH 7.4 were chosen for subsequent experiments.

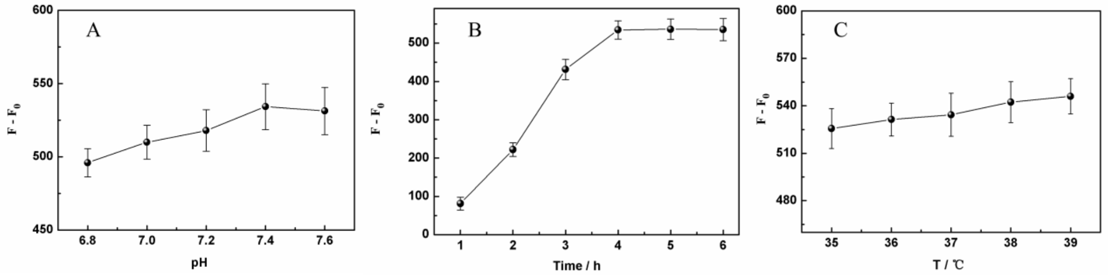

Figure S1. (A) Effect of the incubation time, (B) pH and (C) temperature, on the fluorescence intensity responding of 1.0 $\times 10^{-8}$ M GSH in HB solution.

Specificity of the Assay

To test the selectivity of this method, the fluorescence response to GSH, glucose, lysine, and Cys at a concentration of 5.0 $\times$ 10^{-8} M was investigated. It is clear that only Cys and GSH showed significantly higher fluorescence intensity (Figure S2). These results show that the proposed method is practical for detecting the thiols in the presence of other interferences, implying fine selectivity and an interesting potential for the analysis of this species of thiol (typically named GSH) in complex environments, such as biological samples.

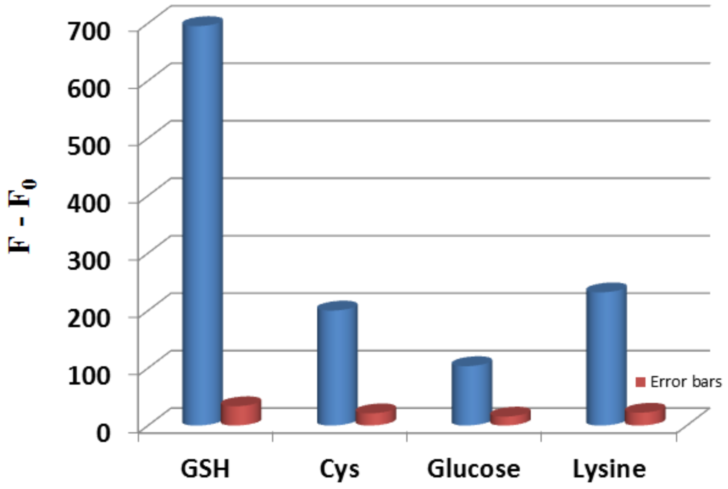

Figure S2. Comparison of the fluorescence intensity produced by 5.0 $\times 10^{-8}$ M GSH, Cys, Glucose, and Lysine, where F represents fluorescence intensitiy of amplification products; F_0 represents fluorescence intensitiy in the absence of GSH. Error bars were estimated from three replicate measurements.

Preparation of PCGs-MSNs

MSNs were synthesized with some modifications on the basis of reference.[S1] 10.0 L ultrapure water, 34.7 g $TEAH_3$ and 192.0 g CTATos were stirred at 80 ℃ for 100 min. Then 1458.0 g TEOS was rapidly added into the surfactant solution only after the surfactant was completely dissolved in water containing the small organic amine (SOA). This mixture (molar composition, SiO_2 : CTATos : SOA : H_2O =1.0 : 0.06 : 0.026 : 80.0) was stirred at 80 ℃ for 2 hours. These MSNs were filtered, swashed, and dried in the oven at 95 ℃ for 20 hours. Then 6 g MSNs were dispersed in 600 ml anhydrous ethanol, APTES was added and stirred for 6 h at 37 ℃. The prepared product was filtered and washed with ethanol and dried at 60 ℃ to obtain PCGs-MSNs.

The fluorescence intensities of time course of the HepG2 cells

The cells were incubated with 600 μL culture medium containing the core-$H_1H_2H_3$ probes probes, then were washed three times with phosphate buffered solution (pH 7.4). The fluorescence intensity was attained the maximum at 240 min in

Figure S3. The fluorescence intensitiy changed hardly after 240 min.[S2, S3]

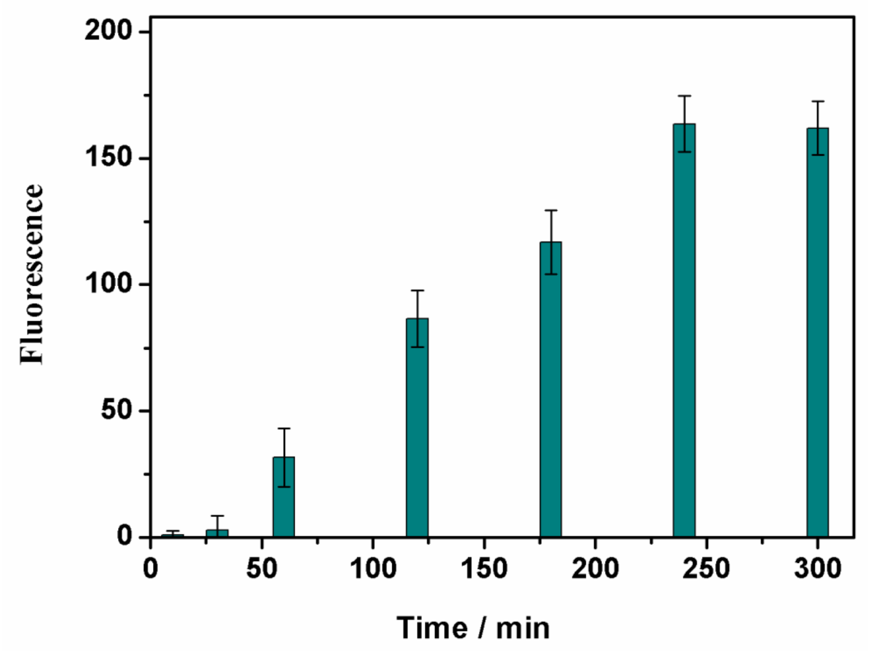

Figure S3 The fluorescence intensities of time course of the HepG2 cells by the core-HCR method catalyzed via a thiol-disulfide exchange reaction.

References

S1. Kun Z. *et al.* Facile large-scale synthesis of monodisperse mesoporous silica nanospheres with tunable pore structure. *J. Am. Chem. Soc.* **135**, 2427-2430 (2013).

S2. Li L. *et al.* Two-color imaging of microRNA with enzyme-free signal amplification via hybridization chain reactions in living cells. *Chem. Sci.*, **7**, 1940-1945 (2016).

S3. Wu Z. *et al.* Electrostatic Nucleic Acid Nanoassembly Enables Hybridization Chain Reaction in Living Cells for Ultrasensitive mRNA Imaging. *J. Am. Chem. Soc.*, **137**, 6829-6836 (2015).
